# 6‐Nitrodopamine Potentiates Catecholamine‐Induced Ca^2+^_i_ Release in Human Aortic Smooth Muscle and Modulates Vascular Smooth Muscle Contractility

**DOI:** 10.1111/bcpt.70224

**Published:** 2026-03-18

**Authors:** José Britto‐Júnior, Antonio Tiago Lima, Shuaihua Qiao, Hou Fong Tang, Valerie Cardenas, Edson Antunes, Gilberto De Nucci, Albert Ferro

**Affiliations:** ^1^ Faculty of Life Sciences and Medicine School of Cardiovascular and Metabolic Medicine & Sciences, British Heart Foundation Centre of Research Excellence, King's College London UK; ^2^ Faculty of Medical Sciences, Department of Pharmacology University of Campinas (UNICAMP) Campinas Brazil; ^3^ Department of Pharmacology, University of São Paulo (USP) Institute of Biomedical Sciences (ICB) São Paulo Brazil

**Keywords:** cAMP, cyclase associated proteins, hypertension, nitrocatecholamine, synergism

## Abstract

**Background:**

In vascular smooth muscle, rises in intracellular calcium [Ca^2+^]_i_ drive contraction downstream of α_1_‐adrenoceptor activation via IP3. Endothelium‐derived 6‐nitrodopamine (6‐ND) augments cardiac catecholamine actions; however, its effect on [Ca^2+^]_i_ is unknown. We hypothesized that 6‐ND would increase intracellular [Ca^2+^]_i_ and potentiate catecholamine‐induced [Ca^2+^]_i_ signalling in vascular smooth muscle cells, with resultant functional effects on vascular tone when measured in vitro.

**Methods:**

Human aortic smooth muscle cells (HASMCs) were loaded with fura‐2 AM (1 μM) and [Ca^2+^]_i_ measured using a CLARIOstar plate reader after addition of Hanks’ balanced salt solution. Rat thoracic aorta rings, with the endothelium removed, were mounted in Krebs–Henseleit baths, and isometric force was recorded via PowerLab.

**Results:**

6‐ND and classical catecholamines evoked concentration‐dependent increases in HASMC Ca^2+^ flux, with 6‐ND displaying the greatest potency. 6‐ND potentiated the increases in HASMC Ca^2+^ flux induced by the classical catecholamines. Tetrodotoxin (TTX) caused a concentration‐dependent inhibition of responses to 6‐ND and dopamine but did not alter noradrenaline‐ or adrenaline‐induced [Ca^2+^]_i_ rise. In endothelium‐denuded aortic rings, 6‐ND potentiated contractions elicited by catecholamines, and this potentiation was abolished by TTX.

**Conclusions:**

6‐ND is a potent modulator of [Ca^2+^]_i_ in HASMCs and enhances catecholamine‐driven vasoconstriction. Both effects are blocked by TTX, indicating that 6‐ND modulates voltage‐gated sodium channels upstream of Ca^2+^ entry/release in vascular smooth muscle cells.

## Summary

Blood vessels control blood flow by tightening or relaxing their muscular walls, a process modulated by calcium inside the cells. The endogenous catecholamine 6‐nitrodopamine (6‐ND) increased [Ca^2+^]_i_ levels in human vascular smooth muscle cells at lower concentrations than those triggered by dopamine, noradrenaline, and adrenaline. It also potentiated both the increase in [Ca^2+^]_i_ levels and the contractions of the rat isolated aorta by classical catecholamines, and these effects were blocked by pre‐treatment of either the cells or the tissue with tetrodotoxin. The results indicate that 6‐ND modulates vascular contractility by activating voltage‐gated sodium channels in vascular smooth muscle.

## 1 Introduction

Arterial hypertension is a highly prevalent condition that is a major risk for cardiovascular diseases [1]. Increased peripheral resistance due to heightened resistance arteriole tone is a prominent feature in hypertension, and an increase in intracellular calcium (Ca^2+^_i_) concentration plays a fundamental role in vascular smooth muscle contraction [2], due to the activation of myosin light‐chain kinase and subsequent serine phosphorylation on the regulatory light chain of myosin [3].

Endothelium‐derived 6‐nitrodopamine (6‐ND) is a newly described catecholamine that plays an important role in cardiovascular regulation [4]. It has potent positive chronotropic [5] and inotropic [6] effects, but even more importantly, it shows a remarkable ability to synergize with the classical catecholamines [7]. In pre‐contracted endothelium‐preserved isolated vessels, 6‐ND causes vasorelaxation independently of soluble guanylate cyclase stimulation [8, 9]. Although 6‐ND is a catecholamine, it does not act on adrenergic receptors, as demonstrated in rat isolated atria [10], rat isolated vas deferens [11] and human isolated vas deferens [12]. The use of agarose coupled to 6‐ND for the identification of its receptor in cardiomyocyte membranes has singled out three such proteins, all of which regulate adenylyl cyclase, namely cyclase‐associated protein 1 (CAP1), cyclase‐associated protein 2 (CAP2) and stromal interaction protein 1 (STIM1) [13].

Inhibitors of the sympathetic nervous system are widely used in the treatment of hypertension [14]. Activation of α_1_‐adrenoceptors by noradrenaline increases inositol triphosphate (IP3) generation, resulting in Ca^2+^ mobilization from intracellular stores via the mediation of Gq/G11 [15], in turn leading to store depletion and activation of store‐operated calcium channels [16]. Thus, investigating the interaction between novel catecholamines and vascular smooth muscle calcium metabolism is therefore of importance, not only to better understand the underlying physiological mechanisms of vascular tone control but also to identify novel cardiovascular therapeutic targets. Since 6‐ND potentiates the action of the classical catecholamines in several tissues [4], we hypothesized that 6‐ND would increase intracellular [Ca^2+^]_i_ and potentiate catecholamine‐induced [Ca^2+^]_i_ signalling in vascular smooth muscle cells, with resultant functional effects on vascular tone when measured in vitro. We therefore investigated the changes in Ca^2+^_i_ concentration induced by 6‐ND in human aortic smooth muscle cells (HASMCs) and whether this novel catecholamine modulates the changes in Ca^2+^_i_ concentrations induced by dopamine, noradrenaline and adrenaline. We also evaluated the interaction of 6‐ND with the classical catecholamines in the rat isolated endothelium‐denuded aorta.

## 2 Methods

### 2.1 Cell Culture

HASMCs at passage 4 or 6 were cultured in smooth muscle cell medium (SMCM, Sciencell) and supplemented with smooth muscle cell growth supplement (Sciencell), 5% fetal bovine serum and penicillin/streptomycin solution (20 units/mL). The Ca^2+^ assay was conducted on live cells cultured in TC‐treated, clear‐bottom, black 96‐well plates. Cells were seeded at a density of > 5 × 10^4^ cells/100 μL in sterile plates and incubated for 48 h at 37°C in a 95% air/5% CO_2_ atmosphere before the experiments. The study was conducted in accordance with the Basic & Clinical Pharmacology & Toxicology policy for experimental and clinical studies [17].

### 2.2 Measurement of [Ca^2+^]_i_

For most experiments, HASMCs were loaded with 1 μM fura 2 AM in SMCM for 45 min at 37°C [18]. After incubation, the cells were washed, and 100 μL Hanks’ balanced salt solution (HBSS) was added, composed of: sodium chloride (NaCl) 118.4 mM, potassium chloride (KCl) 4.7 mM, magnesium sulfate (MgSO_4_) 1.2 mM, sodium dihydrogen phosphate (NaH₂PO₄) 1.2 mM, sodium bicarbonate (NaHCO_3_) 25 mM, glucose 11 mM and **calcium chloride (CaCl₂)** 1.26 mM. The experiments were performed in a thermostatically controlled (37°C) BMG Labtech CLARIOstar Microplate Reader (Aylesbury, UK). Excitation was set at 340 and 380 nm, with emission detected at 510 nm, and the 340:380 emission ratio was used as an index of [Ca^2+^]_i_. The [Ca^2+^]_i_ levels were monitored for 4.5 min following agonist application. In each batch of cells, ionomycin (1 μM) was used as a positive control to assess cell functionality.

### 2.3 6‐ND–, Dopamine‐, Noradrenaline‐ and Adrenaline‐Induced [Ca^2+^]_i_ Increases

Single concentrations of 6‐ND (1 pM to 100 nM), noradrenaline (100 pM to 1 μM), adrenaline (10 nM to 1 μM) and dopamine (100 pM to 100 nM) were added to individual wells of the plates containing HASMCs. Each concentration was applied to a separate well. To evaluate the effects of 6‐ND, noradrenaline, adrenaline and dopamine on [Ca^2+^]_i_, both the peak increase and area under the curve (AUC) of [Ca^2+^]_i_ were analysed.

### 2.4 Effects of 6‐ND, Dopamine, Noradrenaline and Adrenaline on [Ca^2+^]_i_ Increases in HBSS Nominally Calcium‐Free

Single concentrations of 6‐ND (100 pM to 30 nM), noradrenaline (10 nM to 100 nM), adrenaline (100 nM to 1 μM) and dopamine (1 nM to 100 nM) were added to individual wells of the well plates containing HASMCs. Experiments were conducted in HBSS under two different conditions: in the presence and absence of extracellular calcium. The impact of nominally calcium‐free conditions on [Ca^2+^]_i_ responses was assessed to determine whether extracellular calcium influx contributes to the observed effects.

### 2.5 6‐ND Interaction With Dopamine‐, Noradrenaline‐ and Adrenaline‐Induced [Ca^2+^]_i_ Increase

Single concentrations of 6‐ND (1 pM), noradrenaline (30 pM), adrenaline (30 pM) and dopamine (30 pM) were added to the well plates containing HASMCs. Additionally, the effect of pre‐incubation with 6‐ND (1 pM, 1 min) on noradrenaline‐, adrenaline‐ and dopamine‐induced calcium responses was assessed.

### 2.6 Interaction of Noradrenaline, Adrenaline and Dopamine on [Ca^2+^]_i_

Single concentrations of noradrenaline (30 pM), adrenaline (30 pM) or dopamine (30 pM) were added to the well plates containing HASMCs. The comparisons included noradrenaline versus dopamine, noradrenaline versus adrenaline and adrenaline versus dopamine at the specified concentration (30 pM).

### 2.7 The Effect of Tetrodotoxin on [Ca^2+^]_i_

Single concentrations of tetrodotoxin (TTX; 10 nM) were added to well plates containing cultured cells. Additionally, to assess the inhibitory effect of TTX, cells were pre‐treated with TTX (10 nM or 100 nM) before stimulation with 6‐ND (30 nM).

### 2.8 Animals

Adult male Wistar rats (280–320 g) were obtained from CEMIB‐UNICAMP and ANILAB (São Paulo, Brazil). All experimental protocols were approved by the UNICAMP Ethics Committee (Protocol N^o^. 5942‐1/2022), following Brazilian and ARRIVE guidelines [19]. Animals were housed in ventilated cages (three per cage) under controlled conditions (55% ± 5% humidity, 24°C ± 1°C, 12‐h light–dark cycle) and provided with filtered water and standard rodent food ad libitum. As this study was exploratory in nature rather than intended to compare responses between different groups, female animals were not included to reduce the total number of animals used. This decision was made in accordance with ethical considerations aimed at minimizing the use of animals in research [19, 20].

### 2.9 Preparation of Rat Isolated Thoracic Aorta Rings

Euthanasia was performed using an overdose of isoflurane, with animals exposed to a concentration greater than 5% until respiration ceased for at least 1 min. Exsanguination was conducted to confirm euthanasia. The thoracic aorta was isolated and cut into 5‐mm rings, which were mounted between metal hooks in 10 mL organ baths containing heated (37°C, using a heated circulator [PolyScience, IL, USA]), oxygenated (95% O_2_/5% CO_2_) Krebs–Henseleit solution (KHS) of the following composition (in mM): NaCl 118.3, KCl 4.7, MgSO_4_ 1.2, NaHCO_3_ 25.0, KH_2_PO_4_ 1.2, glucose 11.1 and CaCl_2_ 2.5, connected to a PowerLab Acquisition System (ad Instruments, Sydney, Australia). The aorta was equilibrated for 1 h, following which contraction was induced by KCl (80 mM) at the start and at the conclusion of the experiment to assess tissue viability. The endothelium was removed by gently eroding the inner layer using a metallic forceps. To confirm removal of the endothelium, pre‐contraction was performed with phenylephrine (1 μM), following which acetylcholine (10 μM) was added, and rings that showed no relaxation were considered to have a denuded endothelium [21].

### 2.10 The Effect of 6‐ND on the Thoracic Aortic Ring Contractions Induced by Dopamine, Noradrenaline and Adrenaline

To assess the effect of 6‐ND on the concentration‐dependent contractions of isolated rat thoracic aorta, rings were prepared without endothelium and placed in organ baths. Contractions were induced by increasing concentrations of noradrenaline (100 pM–100 μM), adrenaline (100 pM–100 μM) or dopamine (100 pM–100 μM). Pre‐incubation with 6‐ND at concentrations of 1, 10 or 100 pM was carried out for 30 min before the administration of each agonist. The concentration‐response curves to dopamine, noradrenaline and adrenaline were constructed for both control rings (no pre‐treatment) and rings pre‐treated with 6‐ND at the indicated concentrations.

### 2.11 The Effect of TTX on the Potentiation Induced by 6‐ND on the Thoracic Aortic Ring Contractions Induced by Dopamine, Noradrenaline and Adrenaline

To evaluate the effect of 6‐ND on concentration‐dependent contractions of isolated rat thoracic aorta in the presence of TTX, thoracic aortic rings were prepared without endothelium. Contractions were induced by cumulative concentrations of noradrenaline (100 pM–100 μM), adrenaline (100 pM–100 μM) or dopamine (100 pM–100 μM). In one experimental group, rings were pre‐incubated with TTX (1 μM) for 30 min before the administration of each agonist. In another set of experiments, rings were pre‐incubated with both TTX (1 μM) and 6‐ND (100 nM) for 30 min before the agonist administration.

### 2.12 Effect of Prazosin on 6‐ND– and Noradrenaline‐Induced Contractions in Endothelium‐Denuded Thoracic Aorta

To evaluate the involvement of α1‐adrenoceptors in 6‐ND and noradrenaline‐induced contractions, endothelium‐denuded thoracic aortic rings were pre‐incubated with the α1‐adrenoceptor antagonist prazosin (10 nM) for 30 min before construction of cumulative concentration–response curves to 6‐ND or noradrenaline. Responses were compared with time‐matched control rings without prazosin.

### 2.13 Data Analysis

AUC for [Ca^2+^]_i_ was calculated using Excel, and peak increase was identified by analysing the maximal change between consecutive data points. Nonlinear regression analysis to determine pEC_50_ was performed using GraphPad Prism (GraphPad Software, version 10), with the constraint that F = 0. All concentration–response data were evaluated for a fit to a logistic function in the form: *E* = *E*_max_/([1 + (10^*c*/10^*x*)]^*n* + *F*), where *E* represents the increase in contractile response induced by the agonist, *E*_max_ is the maximum effect of the agonist, c is the logarithm of the concentration of the agonist that produces 50% of *E*_max_, *x* is the logarithm of the drug concentration, *n* is the exponential term that defines the slope of the concentration–response curve, and *F* is the response observed in the absence of the added drug. In all experiments, Student's two‐tailed unpaired *t* test was used to compare differences between two groups, and one‐way ANOVA followed by the Newman–Keuls post hoc test was applied when more than two groups were analysed. A *p* value of less than 0.05 was considered statistically significant. Data are expressed as mean ± SEM Since the study was exploratory in nature, the *p* values should be considered descriptive [22].

### 2.14 Reagents

6‐Nitrodopamine (6‐ND; N493720) was acquired from Toronto Research Chemicals (Ontario, Canada). Adrenaline (21245), dopamine (36532), noradrenaline (35580) and TTX (14964) were purchased from Cayman Chemical Co (Michigan, USA). Fura‐2 AM (1051E) used for [Ca^2+^]_i_ measurement was obtained from Ion Biosciences (San Marcos, USA). CaCl₂, glucose, MgSO₄, KCl, potassium phosphate monobasic (KH₂PO₄), NaHCO₃, NaCl and NaH₂PO₄ used to prepare KHS and HBSS were obtained from Merck KGaA (Darmstadt, Germany).

## 3 Results

### 3.1 Effects of 6‐ND, Noradrenaline, Adrenaline and Dopamine on [Ca^2+^]_i_ in HASMCs

**FIGURE 1:**
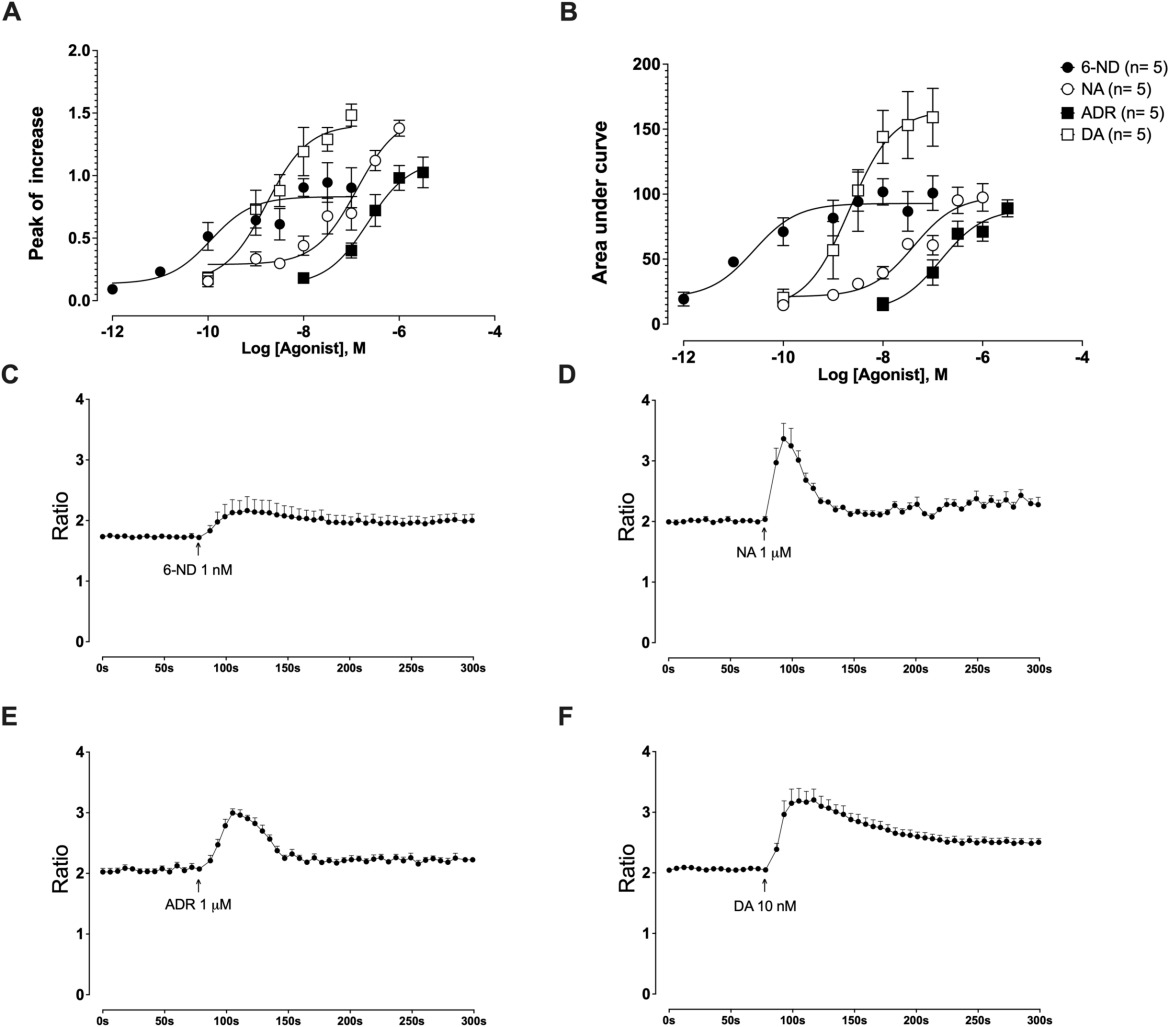
Effect of 6‐nitrodopamine (6‐ND), noradrenaline (NA), adrenaline (ADR) and dopamine (DA) on intracellular calcium ([Ca^2+^]_i_) in human aortic smooth muscle cells. Panels A and B show the concentration‐dependent increases in [Ca^2+^]_i_ induced by 6‐ND, NA, ADR and DA, as measured by the peak increase and area under the curve, respectively. Panels C to F show accumulated traces of [Ca^2+^]_i_ increase at 6‐ND 1 nM (C), NA 1 μM (D), ADR 1 μM (E) and DA 10 nM (F). Data are expressed as mean ± SEM.

The 6‐ND, noradrenaline, adrenaline and dopamine induced concentration‐dependent increases in Ca^2+^ flux in HASMCs are shown in Figure 1A,B. As presented in Table 1, 6‐ND demonstrated significantly higher potency than each of noradrenaline, adrenaline and dopamine when assessed either according to peak increase or AUC (*p* < 0.05). About *E*_max_ for peak increase, 6‐ND produced a similar maximal effect to adrenaline, whereas noradrenaline and dopamine induced greater *E*_max_ responses. For AUC, the response was comparable between 6‐ND and noradrenaline, whereas adrenaline elicited a lower response and dopamine elicited the highest response. Accumulated traces for [Ca^2+^]_i_ increase are shown for 6‐ND (1 nM; Figure 1C), noradrenaline (1 μM; Figure 1D), adrenaline (1 μM; Figure 1E) and dopamine (10 nM; Figure 1F).

**TABLE 1:** Potency and efficacy of 6‐nitrodopamine (6‐ND) in modulating calcium signalling in HASMCs compared to noradrenaline (NA), adrenaline (ADR) and dopamine (DA).

Catecholamine	Peak		AUC	
	pEC_50_	*E*_max_	pEC_50_	*E*_max_
6‐Nitrodopamine	9.9 ± 0.3^a^ ^–^ ^c^	0.9 ± 0.2^a^, ^c^	10.6 ± 0.4^a^ ^–^ ^c^	100.8 ± 13.3^b^, ^c^
Noradrenaline	6.9 ± 0.2^b^ ^–^ ^d^	1.4 ± 0.1^b^, ^d^	7.4 ± 0.2^b^ ^–^ ^d^	97.5 ± 10.6^b^, ^c^
Adrenaline	6.5 ± 0.3^a^, ^c^, ^d^	1.0 ± 0.1^a^, ^c^	7.0 ± 0.3^a^, ^c^, ^d^	71.4 ± 7.3^a^, ^c^, ^d^
Dopamine	8.8 ± 0.2^a^, ^b^, ^d^	1.5 ± 0.1^b^, ^d^	8.7 ± 0.2^a^, ^b^, ^d^	159.1 ± 22.3^a^, ^b^, ^d^

*Note:* AUC (area under the curve), *E*_max_ (maximum response) and pEC_50_ (concentration at which 50% of *E*_max_ is achieved) were analysed using one‐way ANOVA followed by the Newman–Keuls post hoc test.

^a^ *p* < 0.05 compared with NA.

^b^ *p* < 0.05 compared with ADR.

^c^ *p* < 0.05 compared with DA.

^d^ *p* < 0.05 compared with 6‐ND.

### 3.2 Effects of 6‐ND, Noradrenaline, Adrenaline and Dopamine on [Ca^2+^]_i_ in HASMCs Under Nominally Calcium‐Free Conditions

**FIGURE 2:**
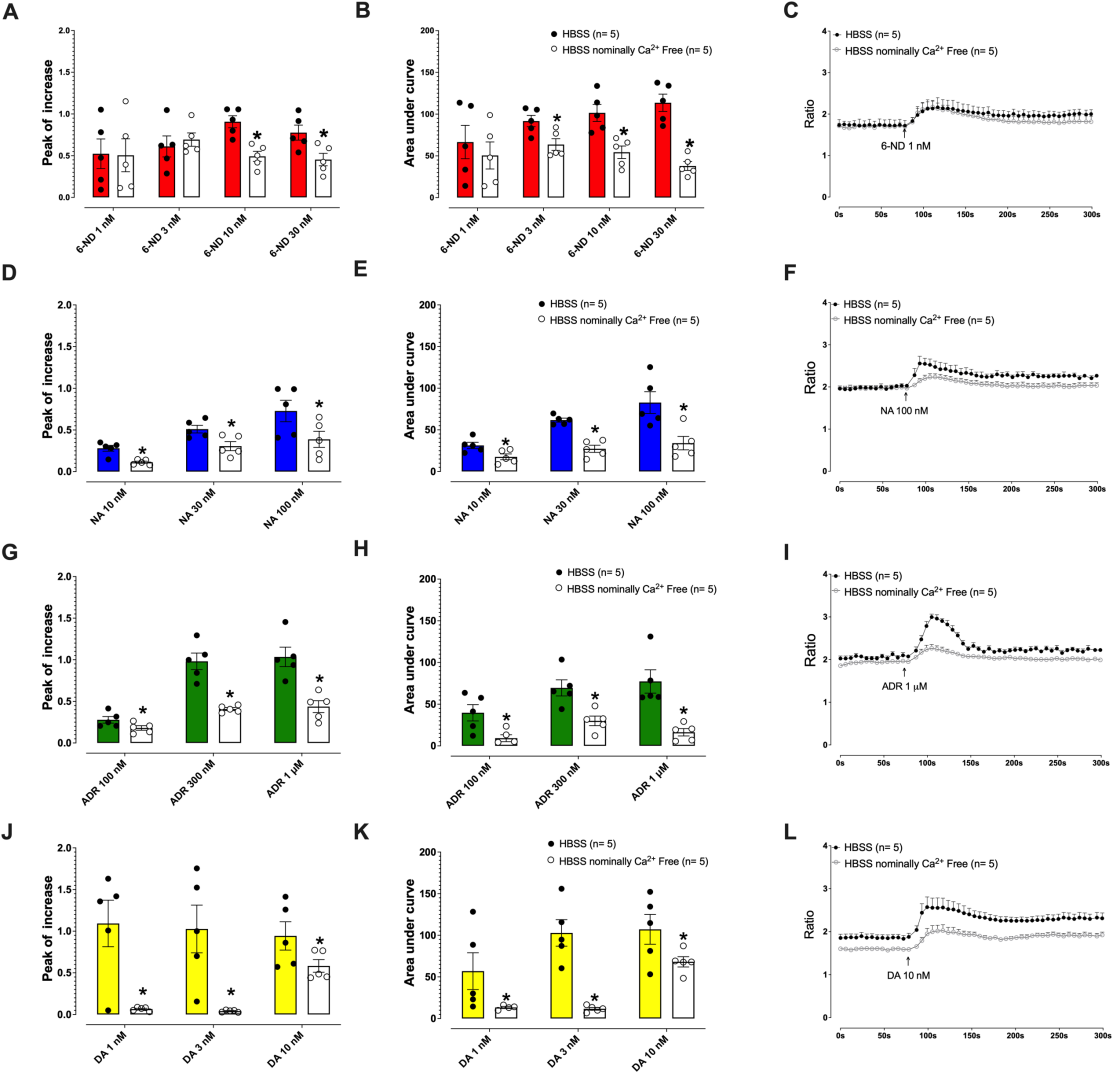
Effect of 6‐nitrodopamine (6‐ND), noradrenaline (NA), adrenaline (ADR) and dopamine (DA) on concentration‐dependent increases in intracellular calcium ([Ca^2+^]_i_) in human aortic smooth muscle cells in Hanks’ balanced salt solution (HBSS), with Ca^2+^ and nominally Ca^2+^‐free. Panels A, B and C show the effect of 6‐ND on [Ca^2+^]_i_, measured by the peak increase and area under the curve (AUC), respectively. Panels D, E and F show the effect of NA on [Ca^2+^]_i_, measured by the peak increase, AUC and accumulated [Ca^2+^]_i_ traces, respectively. Panels G, H and I represent the effect of ADR, and Panels J, K and L represent the effect of DA, on [Ca^2+^]_i_ as measured by these same parameters. *p < 0.05. Data are expressed as mean ± SEM.

In nominally calcium‐free HBSS, 6‐ND exhibited a significantly reduced effect compared to its response in HBSS containing Ca^2+^. As shown in Figure 2A for peak increase, the reduction was observed at 10 and 30 nM (*p* < 0.05). Similarly, AUC (Figure 2B) was significantly lower at 3, 10 and 30 nM (*p* < 0.05). Accumulated [Ca^2+^]_i_ traces for 6‐ND at different concentrations are shown in Figure 2C (1 nM). Noradrenaline gave rise to a lesser [Ca^2+^]_i_ response in calcium‐free HBSS at 100, 300 nM and 1 μM, as demonstrated in Figure 2D–F. Similarly, adrenaline at 10, 30 and 100 nM and dopamine at 1, 3 and 10 nM induced lower [Ca^2+^]_i_ increases in calcium‐free HBSS, as illustrated in Figure 2G–I for adrenaline and Figure 2J–L for dopamine.

### 3.3 Interactions of 6‐ND With Dopamine‐, Noradrenaline‐ and Adrenaline‐Induced Increases in [Ca^2+^]_i_ in HASMCs

**FIGURE 3:**
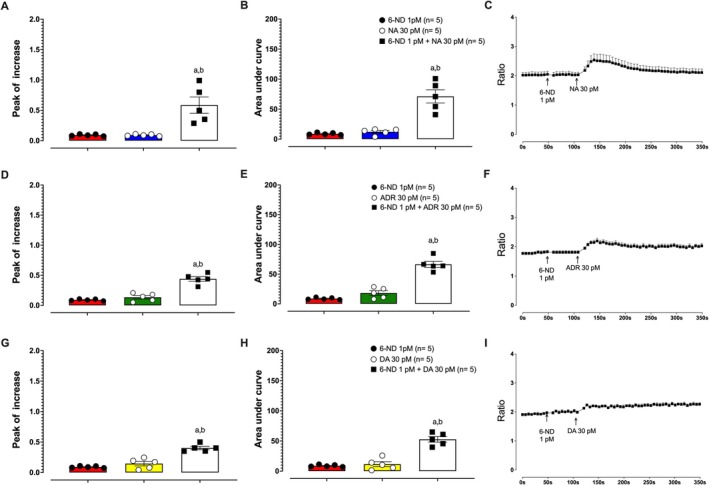
Synergistic effect of 6‐nitrodopamine (6‐ND) on catecholamine‐induced intracellular calcium ([Ca^2+^]_i_) increase in human aortic smooth muscle cells. Panels A, B and C show that pre‐incubation with 6‐ND (1 pM) enhanced the effect of noradrenaline (NA) on [Ca^2+^]_i_ increase, as measured by the peak increase, area under the curve (AUC) and accumulated [Ca^2+^]_i_ traces, respectively. Panels D, E and F demonstrate that pre‐incubation with 6‐ND (1 pM) enhanced the effect of adrenaline (ADR) on [Ca^2+^]_i_ increase, as measured by the peak increase, AUC and accumulated [Ca^2+^]_i_ traces, respectively. Panels G, H and I show that pre‐incubation with 6‐ND (1 pM) similarly enhanced the effect of DA on [Ca^2+^]_i_ increase, as measured by the peak increase, AUC and accumulated [Ca^2+^]_i_ traces, respectively. ^a,b^ *p* < 0.05 compared with 6‐ND (1 pM) and NA, ADR or DA (30 pM). Data are expressed as mean ± SEM.

6‐ND at a concentration of 1 pM had no effect [Ca^2+^]_i_ (Figure 3A–I). Similarly, noradrenaline at 30 pM (Figure 3A–C), adrenaline at 30 pM (Figure 3D–F) and dopamine at 30 pM (Figure 3G–I) had no effect on [Ca^2+^]_i_. On the other hand, pre‐incubation with 6‐ND (1 pM) gave rise to a substantial increase in [Ca^2+^]_i_ in response to noradrenaline (Figure 3A–C), adrenaline (Figure 3D–F) and dopamine (Figure 3G–I), each at 30 pM. Figure S1 shows the effects of noradrenaline, adrenaline and dopamine, each alone or in combination and each at a concentration of 30 pM, on [Ca^2+^]_i_ in HASMCs. None of these catecholamines, either alone or in combination with another, increased [Ca^2+^]_i_.

### 3.4 Effect of 6‐ND on Concentration‐Dependent Contractions of Endothelium‐Denuded Isolated Rat Thoracic Aortic Rings Induced by Noradrenaline, Adrenaline,i and Dopamine

**FIGURE 4:**
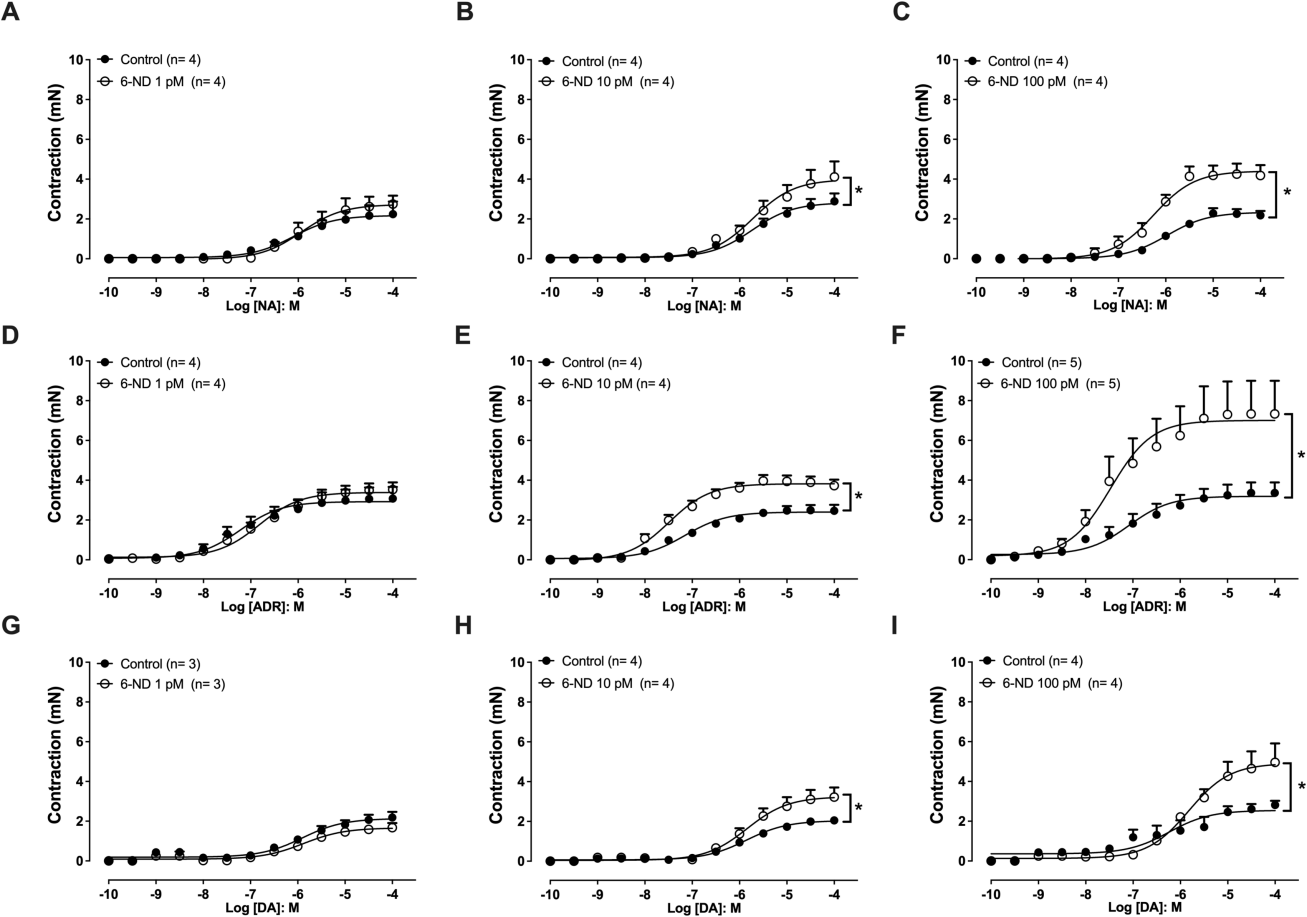
Effect of 6‐nitrodopamine (6‐ND) on concentration‐dependent contractions of isolated rat thoracic aortic rings without endothelium, induced by noradrenaline, adrenaline and dopamine. Panels A to C show the concentration‐dependent contractions induced by noradrenaline (NA; 100 pM–100 μM), in the absence or presence of 6‐ND 1 pM (A), 10 pM (B) and 100 pM (C). Panels D to F illustrate the effect of adrenaline (ADR; 100 pM–100 μM) on contraction, in the absence or presence of 6‐ND 1 pM (D), 10 pM (E) and 100 pM (F). Panels G to I show the effect of dopamine (DA; 100 pM–100 μM), in the absence or presence of 6‐ND 1 pM (G), 10 pM (H) and 100 pM (I). *p < 0.05. Data are expressed as mean ± SEM.

Noradrenaline‐induced concentration‐dependent contractions in isolated rat thoracic aorta rings without endothelium are shown in Figure 4A–C. Pre‐incubation with 6‐ND 1 pM for 30 min had no effect on the contraction‐response curve to noradrenaline (Figure 4A), whereas at 10 pM (Figure 4B) and 100 pM (Figure 4C) significantly increased *E*_max_ in the noradrenaline‐induced contractions. Adrenaline also induced concentration‐dependent contractions (Figure 4D–F), which were not altered by pre‐incubation with 6‐ND 1 pM (Figure 4D), whereas at concentrations of 10 pM (Figure 4E) and 100 pM (Figure 4F) 6‐ND significantly enhanced *E*_max_ for adrenaline‐induced contractions. Similarly, dopamine‐induced concentration‐dependent contractions (Figure 4G–I), which were unaltered by pre‐incubation with 6‐ND 1 pM (Figure 4G), whereas at concentrations of 10 pM (Figure 4H) and 100 pM (Figure 4I) 6‐ND significantly increased *E*_max_ for dopamine‐induced contractions. The data for *E*_max_ and pEC_50_ were described in Table 2.

**TABLE 2:** The potency (pEC_50_) and the maximal responses (*E*_max_) of noradrenaline, adrenaline and dopamine in the rat isolated aorta rings in the absence (control) and presence of 6‐nitrodopamine (6‐ND).

	pEC_50_ (log[M])	*E*_max_ (mN)	*n*
**Noradrenaline**			
Control	6.1 ± 0.2	2.2 ± 0.6	4
6‐ND (1 pM)	5.9 ± 0.2	2.7 ± 0.4	4
Control	5.7 ± 0.1	2.9 ± 0.4	4
6‐ND (10 pM)	5.7 ± 0.1	4.1 ± 0.7*	4
Control	6.0 ± 0.1	2.2 ± 0.2	4
6‐ND (100 pM)	6.2 ± 0.1	4.2 ± 0.5*	4
**Adrenaline**			
Control	7.2 ± 0.2	3.1 ± 0.6	4
6‐ND (1 pM)	6.8 ± 0.1	3.5 ± 0.4	4
Control	7.2 ± 0.1	2.6 ± 0.3	4
6‐ND (10 pM)	7.4 ± 0.1	3.7 ± 0.3*	4
Control	7.1 ± 0.2	3.3 ± 0.5	5
6‐ND (100 pM)	7.5 ± 0.2	7.3 ± 1.6*	5
**Dopamine**			
Control	5.9 ± 0.1	2.2 ± 0.2	3
6‐ND (1 pM)	5.8 ± 0.1	1.8 ± 0.2	3
Control	5.9 ± 0.1	2.1 ± 0.2	4
6‐ND (10 pM)	5.8 ± 0.1	3.2 ± 0.5*	4
Control	6.2 ± 0.2	2.8 ± 0.2	4
6‐ND (100 pM)	5.8 ± 0.1	5.0 ± 0.9*	4

*Note:* The pEC_50_ and *E*_max_ were expressed as mean ± SEM. A two‐tailed unpaired *t*‐test was performed on control versus treated values.

* Represents *p* < 0.05.

### 3.5 Effect of TTX on 6‐ND–, Dopamine‐, Noradrenaline‐ and Adrenaline‐Induced [Ca^2+^i] Increase in HASMCs

**FIGURE 5:**
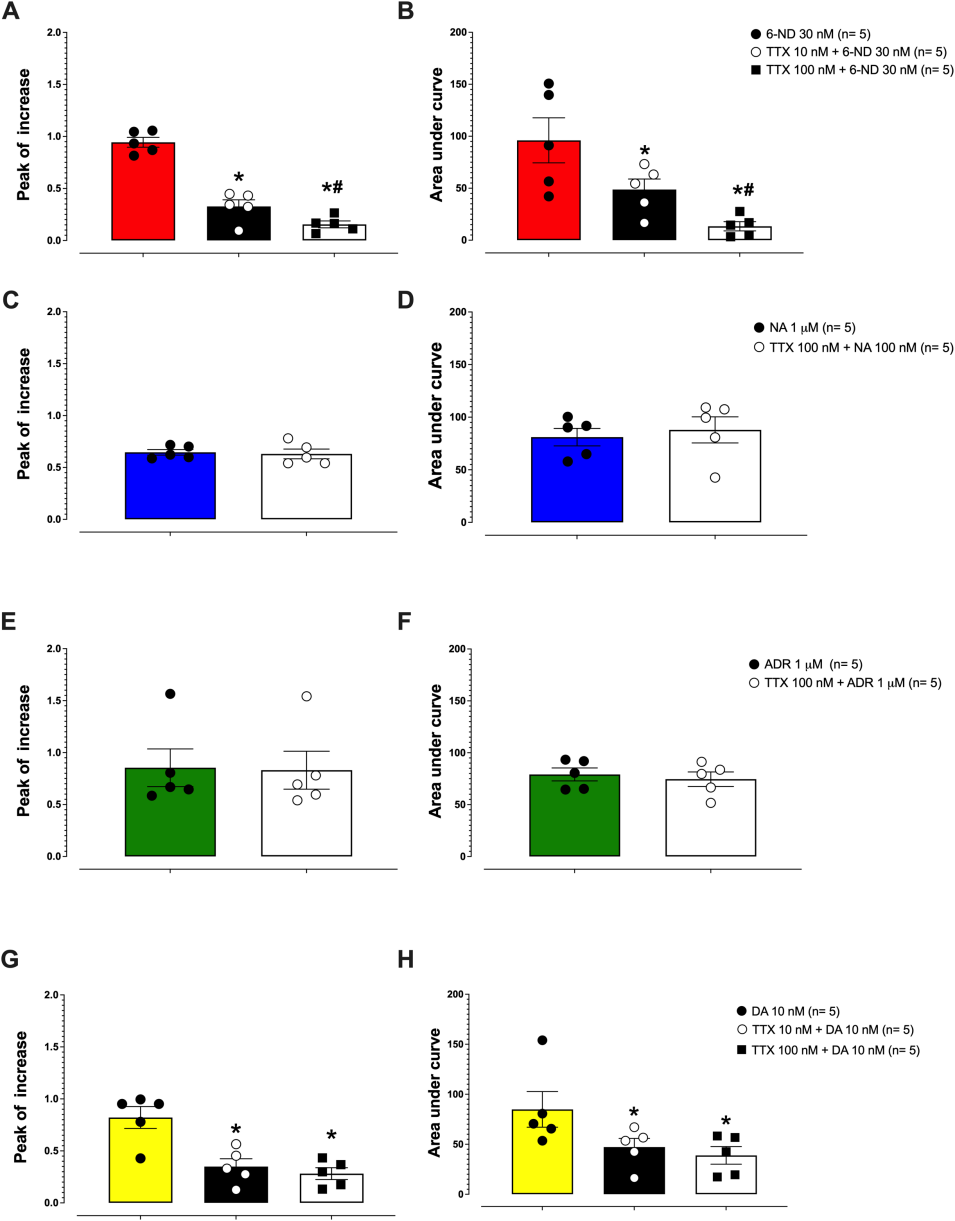
Effect of tetrodotoxin (TTX) on 6‐nitrodopamine (6‐ND), noradrenaline (NA), adrenaline (ADR) and dopamine (DA) induced intracellular calcium ([Ca^2+^]_i_) increase in human aortic smooth muscle cells. Panels A and B show the effect of TTX (10 and 100 nM) on the 6‐ND–induced [Ca^2+^]_i_ increase, measured by peak increase (A) and area under the curve (AUC, B). Panels C and D show the effect of TTX (100 nM) on NA‐induced [Ca^2+^]_i_ increase, measured by peak (C) and AUC (D). Panels E and F present the effect of TTX (100 nM) on ADR‐induced [Ca^2+^]_i_ increase, measured by peak (E) and AUC (F). Panels G and H show the effect of TTX (100 nM) on DA‐induced [Ca^2+^]_i_ increase, measured by peak (G) and AUC (H). Values are expressed as mean ± SEM. *p < 0.05 compared with 6‐ND 30 nM or DA 10 nM; ^#^ *p* < 0.05 compared with TTX 10 nM + 6‐ND 30 nM or TTX 10 nM + DA 10 nM. Data are expressed as mean ± SEM.

TTX at 10 nM had no effect on [Ca^2+^]_i_, as assessed by both peak increase and AUC (*n* = 5; Figure S2). TTX at both 10 and 100 nM reduced the 6‐ND (30 nM)–induced increase in [Ca^2+^]_i_ in a concentration‐dependent manner (Figure 5A,B). In contrast, TTX (100 nM) did not affect the [Ca^2+^]_i_ increase induced by either noradrenaline (1 μM; Figure 5C,D) or adrenaline (1 μM; Figure 5E,F). Tetrotodoxin at 10 and 100 nM reduced the dopamine (DA, 10 nM)**–**induced increase in [Ca^2+^]_i_, but this effect was not concentration‐dependent (Figure 5G,H).

### 3.6 Effect of TTX on the Effect of 6‐ND on Concentration‐Dependent Contractions of Isolated Rat Thoracic Aortic Rings Without Endothelium, Induced by 6‐ND, Noradrenaline, Adrenaline and Dopamine

**FIGURE 6:**
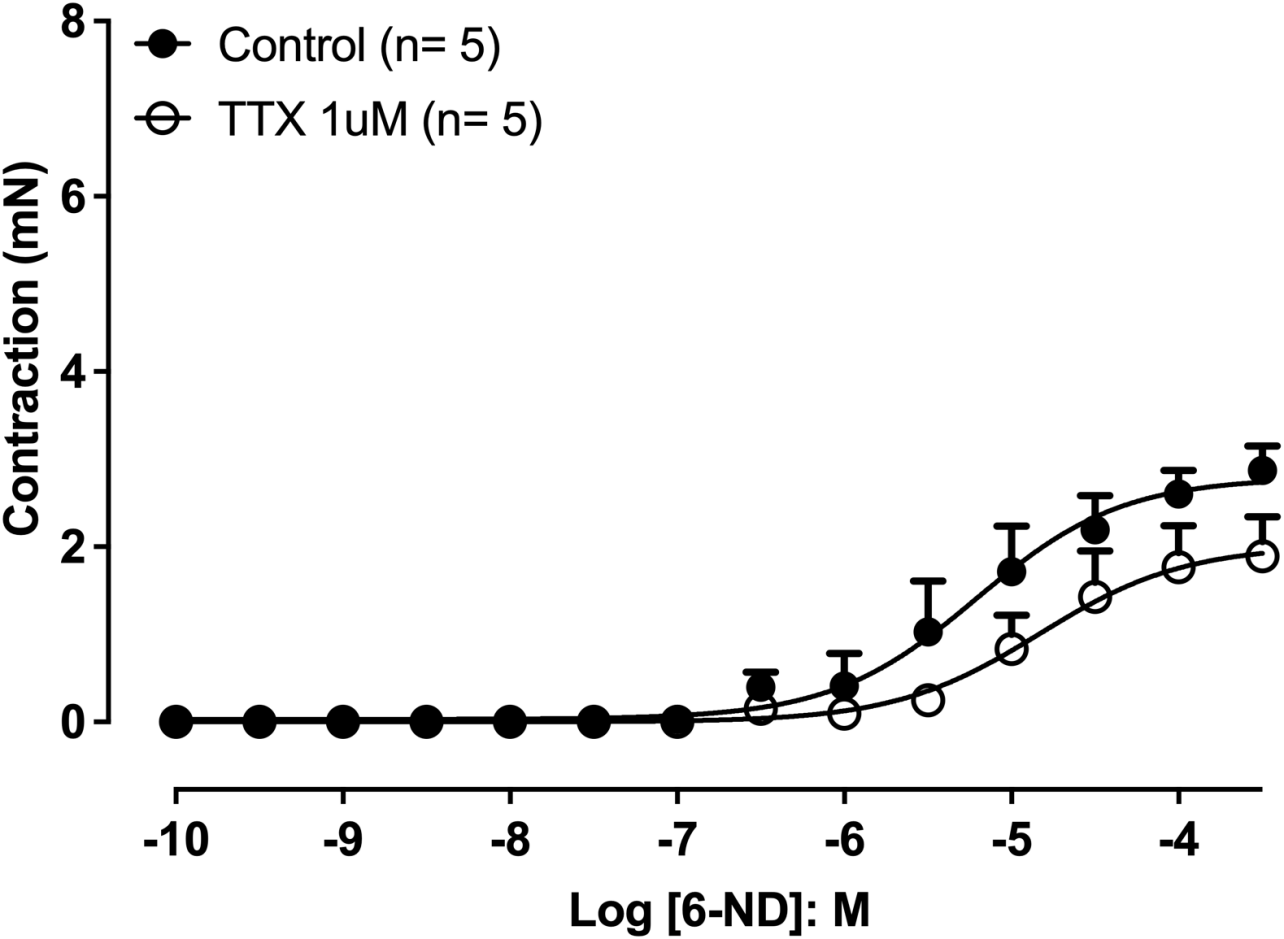
Effect of tetrodotoxin (TTX) on 6‐nitrodopamine–induced contractions in isolated rat thoracic aortic rings without endothelium. Concentration–response curves for 6‐ND were obtained under control conditions and after pre‐incubation with TTX (1 μM). Data are expressed as mean ± SEM.

Pre‐incubation of isolated rat thoracic aortic rings with TTX (1 μM) caused a right shift in the concentration–response curve induced by 6‐ND (Figure **6**) compared with control (pEC_50_ 5.2 ± 0.1 and 4.8 ± 0.2, control and TTX, respectively; *p* < 0.05). In addition, TTX significantly reduced the maximal contractile response (*E*_max_) to 6‐ND (2.8 ± 0.2 mN and 2.0 ± 0.2 mN, control and TTX, respectively; *p* < 0.05). However, pre‐incubation with TTX (1 μM) had no significant effect on the contraction‐response curve induced by noradrenaline (Figure S3A). Similarly, adrenaline‐ and dopamine‐induced concentration‐dependent contractions (Figure S3B and S3C, respectively) were not significantly altered by the presence of TTX. Figure S3D–F illustrates the effect of 6‐ND 100 nM in the presence of TTX (1 μM). Pre‐incubation with 6‐ND (100 nM) in aortic rings treated with TTX did not alter the *E*_max_ for noradrenaline‐induced contractions (Figure S3D), adrenaline‐induced contractions (Figure S3E) and dopamine‐induced contractions (Figure S3F).

### 3.7 Effect of Nominally Ca^2+^‐Free Solution on Agonist‐Induced Concentration‐Dependent Contractions of Isolated Rat Thoracic Aortic Rings Without Endothelium

**FIGURE 7:**
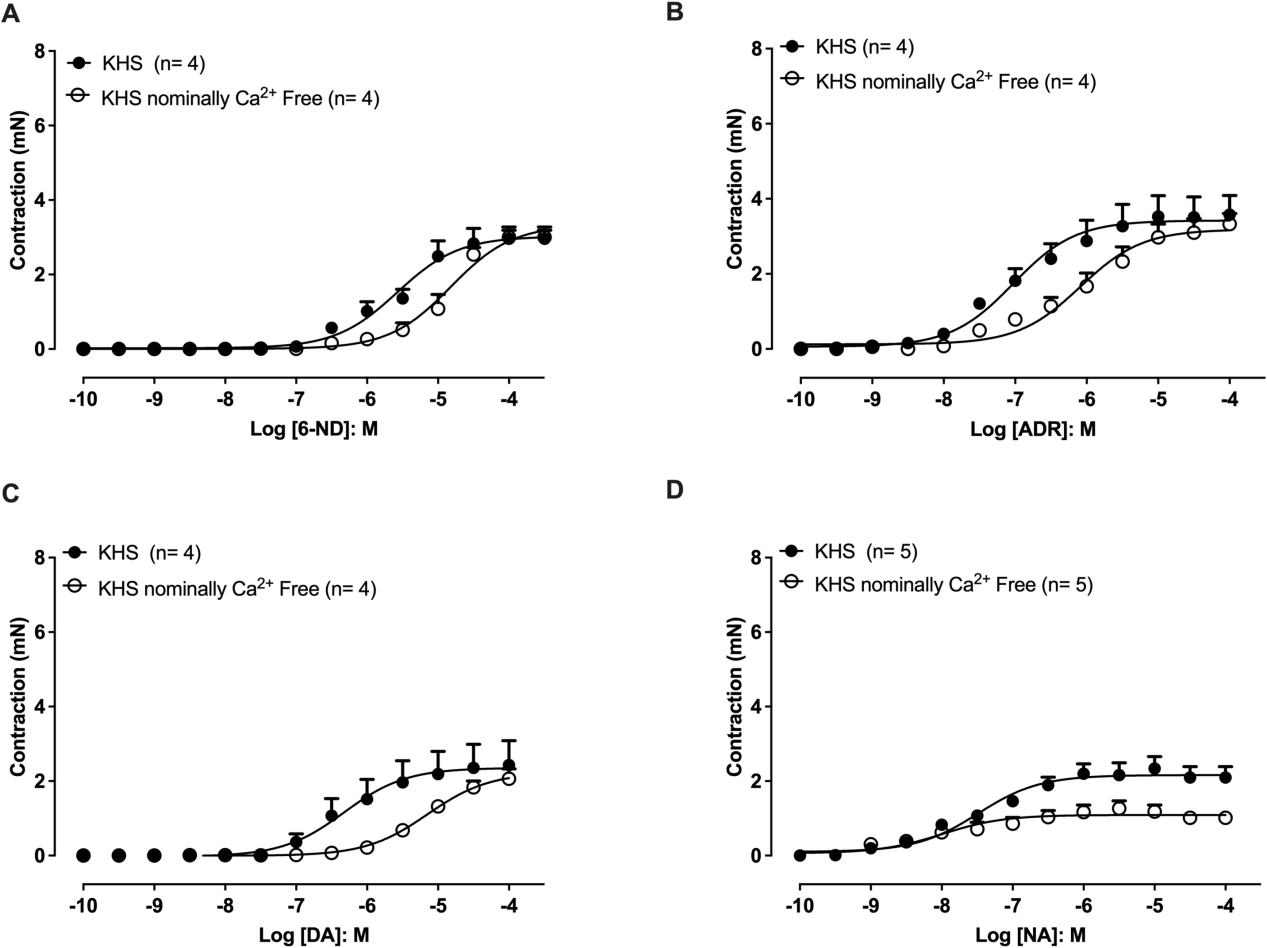
Effect of nominally Ca^2+^‐free solution on agonist‐induced contractions in isolated rat thoracic aortic rings without endothelium. Concentration–response curves for contractions induced by 6‐nitrodopamine (6‐ND; A), adrenaline (ADR; B), dopamine (DA; C) and noradrenaline (NA; D) were obtained in Krebs–Henseleit solution (KHS) containing normal Ca^2+^ or in nominally Ca^2+^‐free KHS. Data are expressed as mean ± SEM.

Incubation of aortic rings in a nominally Ca^2+^‐free KHS caused a right shift of the concentration–response curves induced by 6‐ND compared with control conditions in normal Ca^2+^‐containing KHS (Figure **7**A). Similarly, adrenaline‐ and dopamine‐induced concentration‐dependent contractions also exhibited a right shift in the nominally Ca^2+^‐free solution (Figure **7**B,C, respectively). In contrast, noradrenaline‐induced contractions were attenuated under nominally Ca^2+^‐free conditions, with a reduction in the maximal contractile response compared with control KHS (Figure **7**D).

### 3.8 Effect of Prazosin on 6‐ND– and Noradrenaline‐Induced Contractions in Endothelium‐Denuded Rat Thoracic Aorta

**FIGURE 8:**
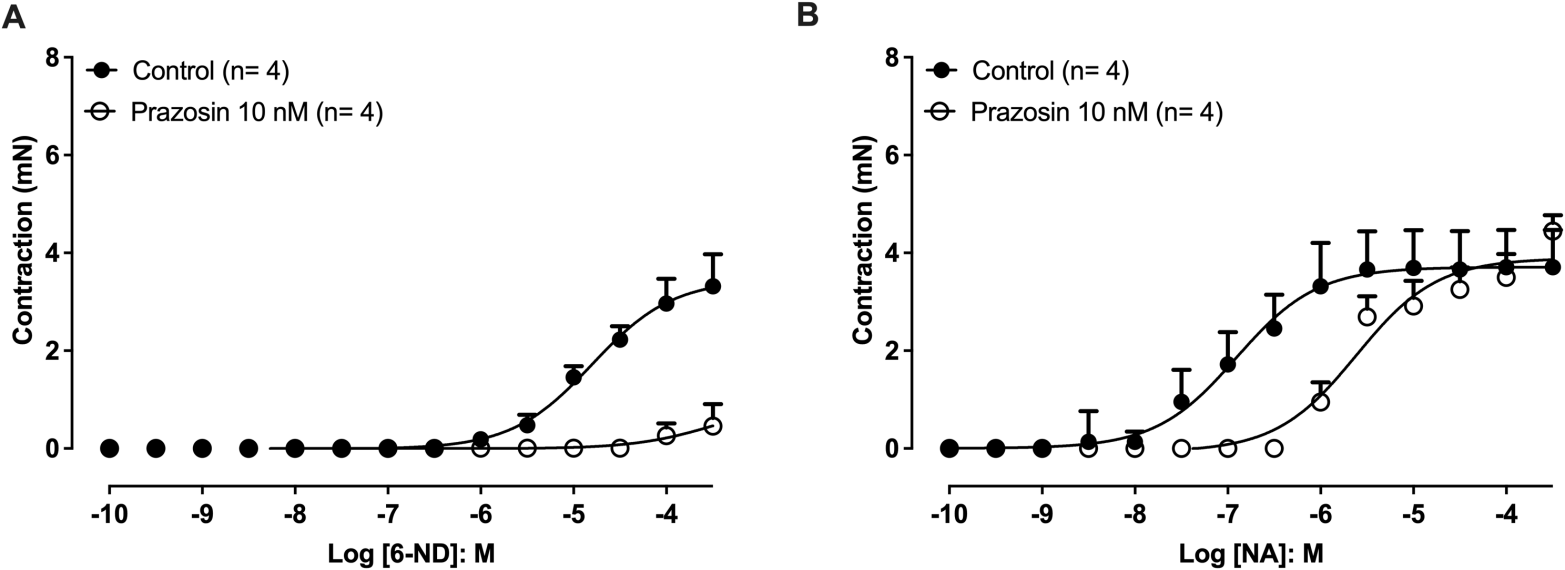
Effect of prazosin on 6‐nitrodopamine (6‐ND) and noradrenaline (NA)–induced contractions in endothelium‐denuded rat thoracic aorta. Concentration–response curves to 6‐ND (A) and NA (B) were obtained in the absence (control) or presence of prazosin (pre‐incubation 30 min, 100 nM), followed by cumulative agonist addition. Data are expressed as mean ± SEM.

Pre‐incubation with prazosin (10 nM) markedly reduced contractions evoked by 6‐ND (Figure 8A). Additionally, prazosin (10 nM) caused a right shift of the noradrenaline concentration–response curve (Figure 8B).

## 4 Discussion

The results clearly demonstrate that 6‐ND is a major modulator of [Ca^2+^]_i_ levels in HASMCs. Indeed, 6‐ND is over 1000 times more potent than noradrenaline and adrenaline in inducing increases in Ca^2+^_i_ and 100 times more potent than dopamine. With a pEC_50_ of approximately 40 pM, 6‐ND is likely the most potent endogenous substance that increases Ca^2+^_i_ levels. Another important observation is the finding that dopamine is 10 times more potent than noradrenaline and adrenaline in inducing increases in Ca^2+^_i_ levels. These findings reinforce the concept that endothelium‐derived dopamine and 6‐ND constitute a major mechanism for control of vascular tone, in contrast to the overrated role attributed to the autonomic nervous system [6].

Adrenaline (1–100 nM) has been shown to induce concentration‐dependent increases in Ca^2+^_i_ in porcine myometrial cells pre‐treated with propranolol [23]. In the absence of extracellular Ca^2+^, adrenaline also significantly increased Ca^2+^_i_; however, the increase was short‐lasting, as reported here for 6‐ND and the other classical catecholamines. Noradrenaline (10 μM) induces a rapid and marked increase in cytosolic calcium in DDT1 smooth muscle cells [24] and at 100 μM increases Ca^2+^_i_ in smooth muscle cells from the rat isolated carotid body [25]. The observed increase induced by noradrenaline in DDT_1_ cells was concentration‐dependent [24], as reported here for HASMCs. In contrast, the increase in Ca^2+^_i_ induced by 6‐ND was not dependent on the concentration, a characteristic also observed for its positive chronotropic [5] and inotropic [6] effects. In contrast to dopamine, noradrenaline and adrenaline, 6‐ND potentiates the increases in Ca^2+^_i_ levels induced by the classical catecholamines, a characteristic also observed regarding the positive chronotropic [7] and inotropic [13] effects of dopamine, noradrenaline and adrenaline.

The increase in [Ca^2+^]_i_ levels induced by noradrenaline in the rat aorta smooth muscle cells [26] was dose‐dependently inhibited by the α_1_‐adrenergic antagonist prazosin, indicating that noradrenaline activates α_1_‐adrenoceptors [26]. In the rat isolated vas deferens [27] and in the rat isolated atria [10], α_1_‐adrenergic antagonists (doxazosin, tamsulosin, alfuzosin, terazosin and prazosin) act as 6‐ND receptor antagonists, since they inhibit the actions induced by 6‐ND at lower concentrations than those required to inhibit the actions of the classical catecholamines. In the present work, prazosin markedly inhibited 6‐ND–induced contractions, supporting the involvement of α_1_‐adrenoceptors in the contractile component of the 6‐ND response. Nevertheless, adrenergic signalling in large vessels can be complex, and α_1_‐adrenoceptor antagonism may reveal additional influences depending on receptor distribution within the vessel wall [28, 29]. Whether the α_1_‐adrenergic antagonists act as selective 6‐ND antagonists in HASMCs is under current investigation.

In the rat isolated aorta, norepinephrine‐induced sustained contraction has been reported to be strongly attenuated by removal of extracellular calcium but is slightly inhibited by organic calcium channel blockers, indicating that the calcium influx is due to both L‐type and non–L‐type calcium channels [30, 31, 32, 33, 34, 35]. The contractions induced by noradrenaline in the rat aorta are significantly inhibited by verapamil [36]. The calcium channel blockers felodipine and nifedipine also attenuated both the stimulated Ca^2+^_i_ and the contraction induced by noradrenaline in the rat isolated aorta [34]. Here, the removal of extracellular calcium reduced both the increases of Ca^2+^_i_ levels in HASCM and the contractions induced by 6‐ND and the classical catecholamines in the rat isolated aorta denuded of endothelium. Calcium channel blockers are often used in the treatment of arterial hypertension [37] and whether they block the increases in Ca^2+^_i_ induced by 6‐ND in HASMCs remains to be investigated.

Cyclase‐associated proteins have been identified as 6‐ND receptors in the cardiomyocytes [17]. The question arises as to whether adenylyl cyclase modulates Ca^2+^_i_ levels or does the latter modulate the former. In nearly all systems, excitation of the cell is followed by a rise in cAMP levels, which is followed by an increased uptake of calcium into the cell [38]. Although Ca^2+^ is not essential for the increase in cAMP levels following cell stimulation, the final physiological response only happens in the presence of calcium. Adenylyl cyclase activity is modulated by physiological transitions in Ca^2+^ levels [39]; in non‐excitable cells, this is associated with store‐operated Ca^2+^ entry [40] induced by either an agonist‐mediated depletion of IP3 or as a consequence of store depletion by ionophore‐driven emptying [41]. cAMP levels can regulate both the channels that allow Ca^2+^ to enter the cytosol [42] and the calcium pumps that extrude it [43]. Agonist–adrenoreceptor interaction can stimulate IP3 production by phospholipase C (PLC), leading to Ca^2+^ release through IP3 receptors [44]. cAMP can modulate both PLC activity [43] and the interaction of receptors with PLC [45]. Thus, the 6‐ND potentiation of Ca^2+^_i_ levels induced by noradrenaline could be due to 6‐ND interaction with the cyclase‐associated proteins mentioned above.

The use of the voltage‐gated sodium‐channel (Na_v_) blocker TTX [46] provoked concentration‐dependent inhibition of Ca^2+^_i_ levels induced by 6‐ND. Na_v_ modulates action potential in neurons, skeletal muscle, cardiac myocytes [47] and cultured HASMCs [48]. Although the absence of *I*_Na_ has been considered a characteristic of vascular smooth muscle cells [49], it is highly expressed in human coronary, aortic and pulmonary myocytes but also in HASMCs [50, 51]. Indeed, primary cultured myocytes derived from human aortic and pulmonary arteries express TTX‐sensitive *I*_Na_ [52], and some Na_v_ regulate Ca^2+^_i_ levels through tonic control of Ca^2+^ influx in human coronary myocytes in primary culture [53].

## 5 Limitations

Although the data corroborate the involvement of Na_v_, the contribution of voltage‐gated calcium channels deserves further investigation. In the rat isolated aorta, the contraction induced by moderate concentrations of KCl is blocked by preincubation with TTX [54], indicating a modulatory role of Na_v_ in vascular smooth muscle contractility. Indeed, in murine mesenteric artery contracted with U‐46619, TTX causes vasorelaxation [55]. Thus, it is possible that 6‐ND causes activation of Na_v_ channels that would lead to an increase in calcium influx in HASMCs. Whether this effect results from a direct action of 6‐ND on Na_v_ or is mediated via a cAMP‐gated Na^+^ channel remains to be further investigated.

Intracellular Ca^2+^ measurements were performed in cultured HASMCs under non–serum‐deprived conditions. Serum deprivation can drive redifferentiation toward a more contractile phenotype [56] and modify Ca^2+^ signalling and L‐type Ca^2+^ channel properties [57]. Whether 6‐ND/catecholamine responses in serum‐deprived cells are modified in this phenotype remains to be established.

## 6 Conclusion

In conclusion, the present work indicates that 6‐ND is a potent modulator of [Ca^2+^]_i_ in HASMCs and enhances catecholamine‐driven vasoconstriction. Both of these effects are blocked by TTX, indicating that 6‐ND modulates Na_v_ channels upstream of Ca^2+^ entry/release in vascular smooth muscle cells. Our findings suggest a novel and important physiological role of 6‐ND in the modulation of catecholaminergic control of vascular tone.

## Author Contributions

Conceptualization: Albert Ferro and Gilberto De Nucci. Data curation: José Britto‐Júnior and Albert Ferro. Formal analysis: José Britto‐Júnior and Albert Ferro. Funding acquisition: Gilberto De Nucci and Albert Ferro. Investigation: José Britto‐Júnior, Antonio Tiago Lima, Shuaihua Qiao, Hou Fong Tang and Valerie Cardenas. Methodology: José Britto‐Júnior, Gilberto De Nucci and Albert Ferro. Project administration: Albert Ferro and Gilberto De Nucci. Supervision: Albert Ferro. Visualization: Albert Ferro. Writing – original draft: José Britto‐Júnior, Edson Antunes, Gilberto De Nucci and Albert Ferro.

## Funding

Sao Paulo Research Foundation (FAPESP) grants 2023/14557‐9 and 2021/14414‐8 (J.B.‐J.), 2021/1393‐6 (A.T.L.), and 2019/16805‐4 (G.D.N.), National Council for Scientific and Technological Development (CNPq) grant 303839/2019‐8 (G.D.N.), and British Heart Foundation Centre for Excellence Award RE/18/2/34213 (A.F.).

## Conflicts of Interest

The authors declare no conflicts of interest.

## Supporting information


**Figure S1** Effect of the interaction of noradrenaline, adrenaline and dopamine on intracellular calcium ([Ca^2+^]_i_) in human aortic smooth muscle cells. Panels A and B show the effects of noradrenaline (NA, 30 pM) and dopamine (DA, 30 pM), either alone or in combination, on [Ca^2+^]_i_, assessed by peak increase and area under the curve (AUC), respectively. Panels C and D show the effects of noradrenaline (30 pM) and adrenaline (ADR, 30 pM), either alone or in combination, on [Ca^2+^]_i_, assessed by peak increase and AUC, respectively, on [Ca^2+^]i increase, in terms of AUC and peak increase, respectively. Panels E and F show the effects of adrenaline (ADR, 30 pM) and dopamine (DA, 30 pM), either alone or in combination, on [Ca^2+^]_i_, assessed by peak increase and AUC, respectively.

**Figure S2** Effect of tetrodotoxin (TTX, 10 nM) and ionomycin (1 μM) on intracellular calcium ([Ca^2+^]_i_) in smooth muscle cells. Incubation of the HASMCs with TTX (10 nM) did not cause significant increases in [Ca^2+^]_i_ levels. Incubation of the HASMCs with ionomycin (1 μM), used as a positive control, increased the [Ca^2+^]_i_ levels.

**Figure S3** Effect of tetrodotoxin (TTX) on effect of the 6‐nitrodopamine (6‐ND) on concentration‐dependent contractions of isolated rat thoracic aortic rings without endothelium, induced by noradrenaline (NA), adrenaline (ADR) and dopamine (DA). Panels A to C show the effect of TTX (1 μM) on the concentration‐dependent contractions induced by NA (A), ADR (B) and DA (C). Panels D to F illustrate the effect of 6‐ND on contractions induced by NA (D), ADR (E) and DA (F) in the presence TTX (1 μM). Data are expressed as mean ± SEM.

## Data Availability

The authors declare that they agree to provide the requested data as required.
