# Supplementary figures and images for: 6‐Nitrodopamine Potentiates Catecholamine‐Induced Ca2+ i Release in Human Aortic Smooth Muscle and Modulates Vascular Smooth Muscle Contractility
Source: Basic Clin Pharmacol Toxicol. 2026 Mar 18;138(4):e70224. doi: 10.1111/bcpt.70224 (PMC12998412; doi:10.1111/bcpt.70224)

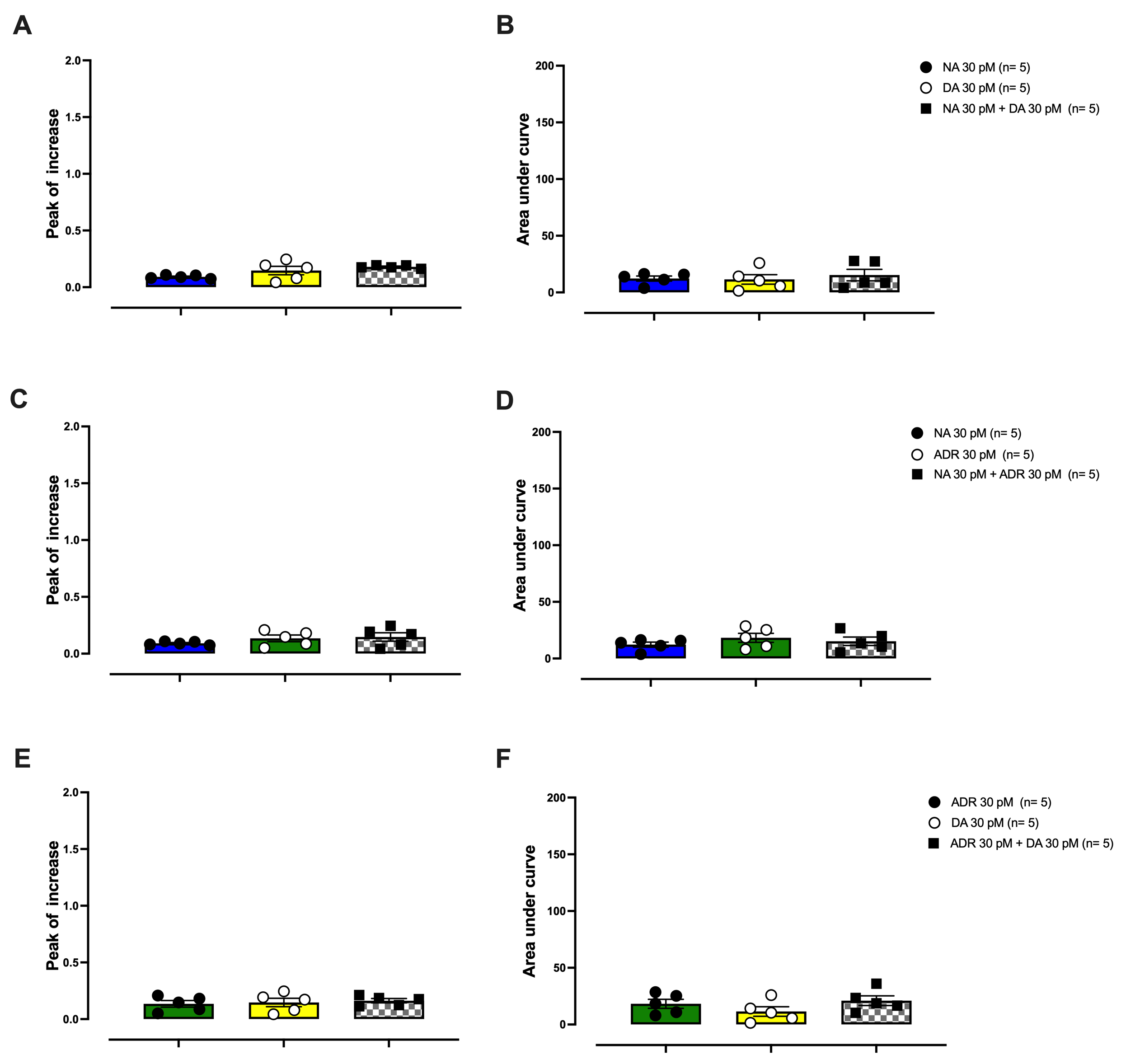

Supplement: Supplementary file 1 — Figure S1 Effect of the interaction of noradrenaline, adrenaline and dopamine on intracellular calcium ([Ca2+]i) in human aortic smooth muscle cells. Panels A and B show the effects of noradrenaline (NA, 30 pM) and dopamine (DA, 30 pM), either alone or in combination, on [Ca2+]i, assessed by peak increase and area under the curve (AUC), respectively. Panels C and D show the effects of noradrenaline (30 pM) and adrenaline (ADR, 30 pM), either alone or in combination, on [Ca2+]i, assessed by peak increase and AUC, respectively, on [Ca2+]i increase, in terms of AUC and peak increase, respectively. Panels E and F show the effects of adrenaline (ADR, 30 pM) and dopamine (DA, 30 pM), either alone or in combination, on [Ca2+]i, assessed by peak increase and AUC, respectively. [file BCPT-138-0-s003.tiff]

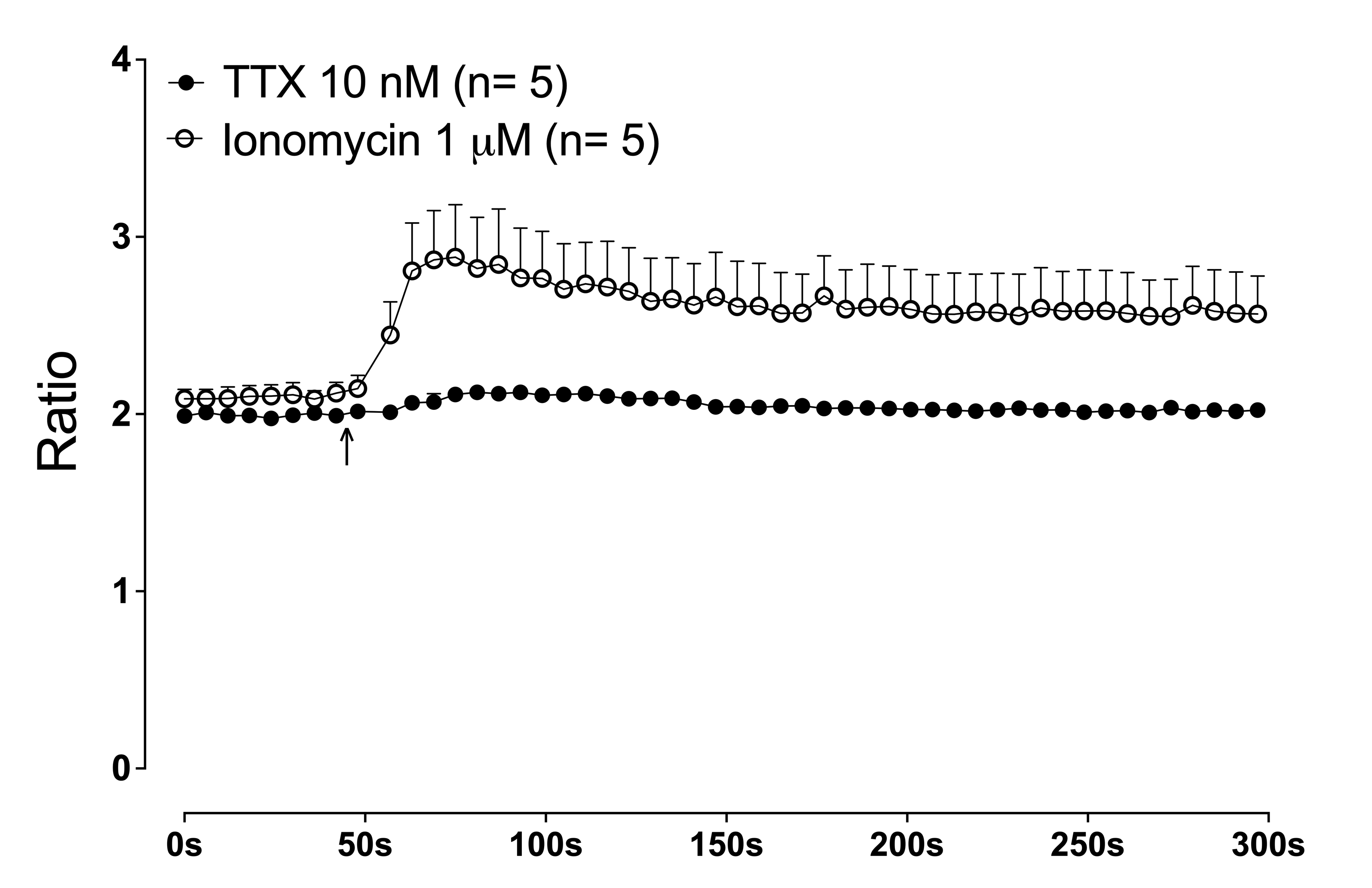

Supplement: Supplementary file 2 — Figure S2 Effect of tetrodotoxin (TTX, 10 nM) and ionomycin (1 μM) on intracellular calcium ([Ca2+]i) in smooth muscle cells. Incubation of the HASMCs with TTX (10 nM) did not cause significant increases in [Ca2+]i levels. Incubation of the HASMCs with ionomycin (1 μM), used as a positive control, increased the [Ca2+]i levels. [file BCPT-138-0-s001.tiff]

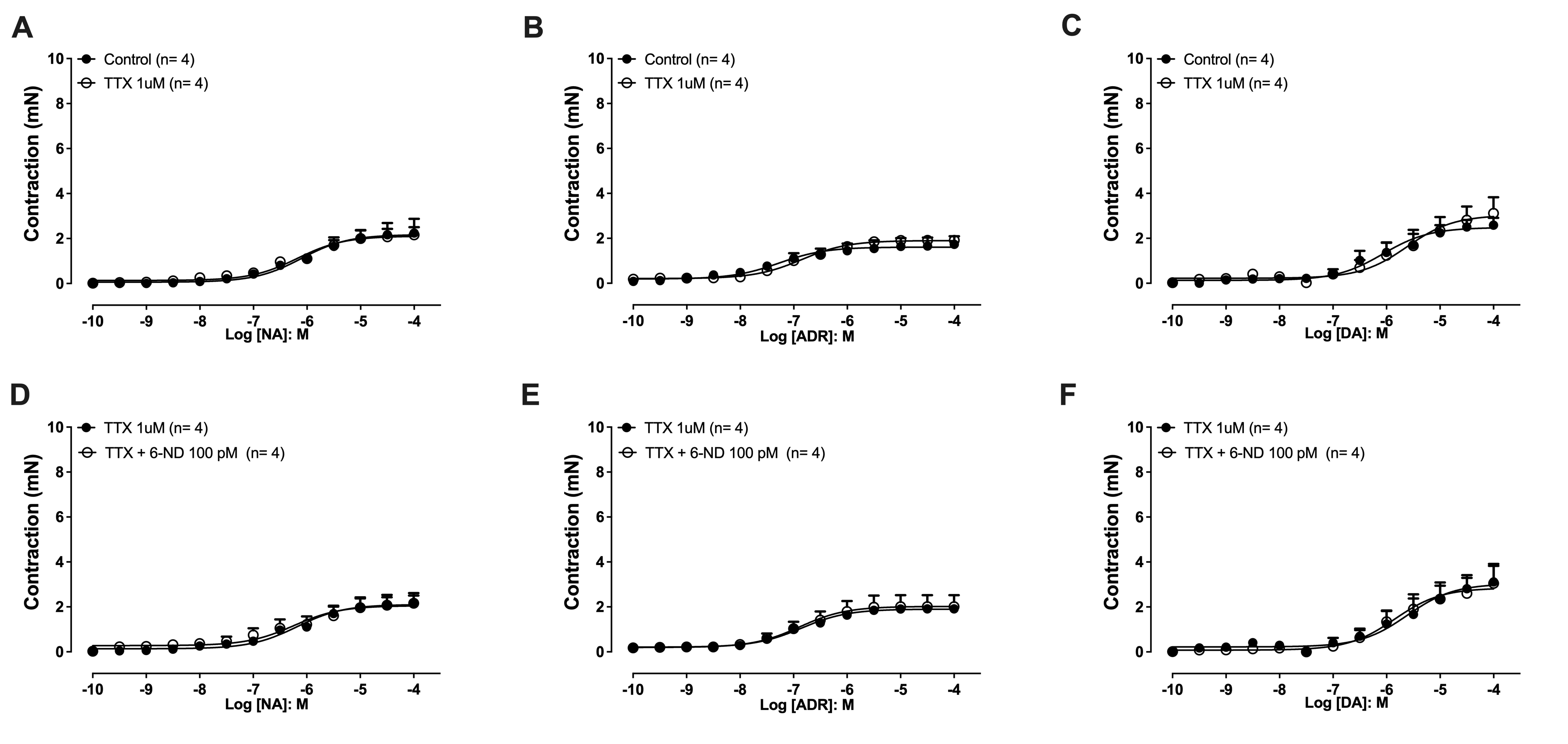

Supplement: Supplementary file 3 — Figure S3 Effect of tetrodotoxin (TTX) on effect of the 6‐nitrodopamine (6‐ND) on concentration‐dependent contractions of isolated rat thoracic aortic rings without endothelium, induced by noradrenaline (NA), adrenaline (ADR) and dopamine (DA). Panels A to C show the effect of TTX (1 μM) on the concentration‐dependent contractions induced by NA (A), ADR (B) and DA (C). Panels D to F illustrate the effect of 6‐ND on contractions induced by NA (D), ADR (E) and DA (F) in the presence TTX (1 μM). Data are expressed as mean ± SEM. [file BCPT-138-0-s002.tiff]
